# Activity of decitabine as maintenance therapy in core binding factor acute myeloid leukemia

**DOI:** 10.1002/ajh.26496

**Published:** 2022-02-22

**Authors:** Jayastu Senapati, Mahran Shoukier, Guillermo Garcia‐Manero, Xuemei Wang, Keyur Patel, Tapan Kadia, Farhad Ravandi, Naveen Pemmaraju, Maro Ohanian, Naval Daver, Courtney DiNardo, Yesid Alvarado, Jeffrey Aldrich, Gautam Borthakur

**Affiliations:** ^1^ Department of Leukemia the University of Texas MD Anderson Cancer Center Houston Texas USA; ^2^ Department of Biostatistics The University of Texas MD Anderson Cancer Center Houston Texas USA; ^3^ Department of Hematopathology The University of Texas MD Anderson Cancer Center Houston Texas USA; ^4^ Department of Internal Medicine The University of Texas MD Anderson Cancer Center Houston Texas USA

## Abstract

**Background:**

Posttherapy measurable residual disease (MRD) positivity in core binding factor acute myeloid leukemia (CBF‐AML) is associated with shorter relapse‐free survival (RFS). Elimination of MRD measured via quantitative reverse transcription polymerase chain reaction (qRTPCR) for disease specific transcripts can potentially lead to better outcomes in CBF‐AML.

**Methods:**

We prospectively monitored the MRD using qRTPCR and flow cytometry on bone marrow samples in patients with newly diagnosed CBF‐AML who received decitabine (DAC) maintenance therapy after fludarabine/cytarabine/G‐CSF (FLAG)‐based induction/consolidation regimen. Negative qRTPCR (CMR) was defined as fusion transcript <0.01%.

**Results:**

Thirty‐one patients with CBF‐AML including 14 with t(8;21) and 17 with inv(16) received parenteral DAC as maintenance therapy. Fifteen patients (48.3%) had completed FLAG‐based induction/consolidation but with positive MRD (0.35%, range = 0.01%–0.91%) (Group 1). Sixteen patients (51.7%) could not complete recommended consolidations with FLAG‐based regimen (due to older age or complications) and were switched to DAC maintenance (Group 2). In Group 2, eight patients (50%) had undetectable MRD (Group 2A) (all had qRTPCR ≤ 0.01%) and the other eight patients (50%) had residual fusion product by qRTPCR (0.1%, range = 0.02%–0.36%) (Group 2B) prior to starting DAC. Amongst the 23 patients who had a PCR ≥ 0.01% before maintenance therapy (Groups 1 and 2B), 12 patients (52%) attained a CMR as their best response (responders). The median pre‐DAC qRTPCR amongst responders were 0.03% compared to 0.14% in nonresponders (*p* = .002). The median estimated molecular RFS amongst responders were 93.9 months. At a median follow‐up of 59.3 months (13.2–106 months) from DAC initiation, 16 patients (51.6%) had to be initiated on a second line of therapy (40%, 25%, and 100% patients, respectively, in Groups1, 2A, and 2B). The median estimated time to new treatment between responders was 112.4 versus 5.8 months in nonresponders (hazard ratio = 0.16, 95% confidence interval = 0.04–0.54); however, there were no difference in overall survival between these groups (*p* = .37).

**Conclusion:**

DAC is an effective maintenance therapy for CBF‐AML patients with persistent fusion transcript at a low level after FLAG‐based regimen. Attainment of CMR with DAC maintenance can lead to long‐term remission in patients who have persistent MRD positive after FLAG‐based regimen or are unable to receive the full course of consolidation therapy.

## 1 INTRODUCTION

Core binding factor (CBF) acute myeloid leukemia (AML) is a subtype of AML, characterized by the presence of t(8;21)(q22;q22) or inv(16)(p13q22)/t(16;16) recurrent translocations, leading to the formation of unique RUNX1/RUNX1T1 (AML1/ETO) or CBFB/MYH112 fusion transcripts, respectively. These cytogenetic aberrations are associated with favorable response and sensitivity to high dose cytarabine based therapy.^1^, ^2^, ^3^, ^4^, ^5^ Fludarabine/cytarabine/G‐CSF (FLAG)‐based regimen has been shown to improve the event‐free survival in CBF‐AML.^6^, ^7^ Despite the high remission rates of > 80% with chemotherapy, disease relapse remains a significant cause of treatment failure with 5‐year overall survival (OS) in the range of 50%–60%.^8^, ^9^, ^10^

The presence of disease defining recurrent fusion transcripts associated with CBF‐AML enable the serial monitoring by real‐time quantitative reverse transcription polymerase chain reaction (qRTPCR) for detection of measurable residual disease (MRD).^11^, ^12^, ^13^ We and others have shown before that post induction monitoring of residual disease with qRTPCR can be useful to identify the patients with higher risk of relapse.^12^, ^14^, ^15^, ^16^ Given the ability to detect early relapse with high sensitivity, disease monitoring using qRTPCR can be helpful in identifying appropriate candidates for further therapy and allogenic stem cell transplantation (allo‐SCT) before frank hematological relapse, as conventionally CBF‐AML patients in first remission are not considered candidates for allo‐SCT.^17^ Studies have shown that high level of PCR persistence after induction or consolidation predisposes the patients to relapse, and 3–4 log reductions from baseline are associated with better outcomes.^11^, ^15^, ^18^, ^19^, ^20^

Gene hypermethylation has been associated with increased risk of relapses in AML.^21^, ^22^ To counter this, hypomethylating agents (HMAs), such as decitabine (DAC) and azacitidine (AZA), have been studied as maintenance agents in AML.^23^, ^24^ Earlier data published by our group, from a smaller cohort of CBF‐AML had shown that HMA maintenance controlled MRD and extended remission in patients who had residual qRTPCR after FLAG‐based induction/consolidation or after ASCT.^25^ The data needed validation in a larger cohort with longer follow‐up and questions remain on the efficacy of HMA maintenance on maintaining MRD negativity in patients who attain negative MRD status after attenuated cycles of a FLAG‐based regimen, and whether such maintenance can extend remission. Here, we further explored the role of DAC maintenance therapy with serial qRTPCR and flow cytometry MRD monitoring in CBF‐AML patients who completed FLAG‐based regimen with persistent positive qRTPCR or those with abbreviated induction/consolidation courses.

## 2 METHODS

We obtained samples from bone marrow (and peripheral blood in patients with long‐term follow‐up) for serial qRTPCR and flow cytometry approximately every 3 months in patients with CBF‐AML who received at least 1 cycle of DAC maintenance for persistent MRD (persistent fusion transcript) or because of inability to complete all planned consolidations of a FLAG‐based regimen (due to age, infectious complications, persistent cytopenia etc.). The sensitivity for transcript qRTPCR detection was between 1 in 10 000 and 1 in 100 000. The methods of the qRTPCR were similar to our previously published work and in line with a Europe against Cancer program.^25^, ^26^ An increase in qPCR from < 0.01% (CMR) was considered as molecular relapse. The planned number of DAC cycles were 12, but investigator discretion was allowed based on qRTPCR status and any other evidence of disease progression or toxicity. We collected the data of baseline hemoglobin, white blood cell counts, platelet counts, bone marrow blasts and cytogenetics (CBF defining and additional cytogenetic abnormalities [ACA]) and the myeloid panel gene mutation results (discussed later).

### 2.1 Treatment regimen

The induction regimen included fludarabine (FL) 30 mg/m^2^ on Days 1–5, cytarabine (A) 2 g/m^2^ IV on Days 1–5, gemtuzumab ozogamicin (GO) 3 mg/m^2^ on Day 1 or idarubicin (Ida) 6 mg/m^2^ on Days 3 and 4, and G‐CSF (G) 5 mcg/kg on Day 1 until neutrophil recovery. The consolidation regimen included FLAG for 3 days with GO on Day 1 in Cycle 2/3 and 5/6 or with Ida (dose: 6 mg/m^2^ on Days 2 and 3 in one postremission cycle), for the planned consolidation of 6 cycles. Thirty‐one patients received monthly maintenance therapy with DAC 20 mg/m^2^ on Days 1–5 every 4–5 weeks based on count recovery and toxicity. The duration of DAC regimen was reduced to 3 days in patients with concerning cytopenias.

### 2.2 Mutation analysis

A multiplex fluorescent‐based PCR analysis followed by capillary electrophoresis for detection of internal tandem duplication (ITD) and tyrosine kinase domain (TKD) mutations in FMS‐like tyrosine kinase 3 (*FLT3)* was performed on DNA isolated from bone marrow aspirate samples as previously described by our group,^27^ with an analytical sensitivity of ~1% mutant DNA in the background of wild‐type DNA. *NRAS*, *KRAS*, *NPM1*, *TP53*, *IDH1*, and *IDH2* and *KIT* mutations in hotspot regions of the coding sequences were identified by next‐generation sequencing using one of three clinical‐grade myeloid gene panels (28‐gene, 53‐gene or 81‐gene) using the Illumina MiSeq (Illumina, Inc.) platform validated at the CLIA‐certified molecular diagnostic laboratory at MDACC as described previously.^28^ A minimum of 250X coverage with a detection sensitivity of ~5% was used for variant calling.

### 2.3 Statistical analysis

Patient and clinical characteristics were summarized using descriptive statistics. OS was calculated from the date of diagnosis to the date of death due to any cause and was censored at the last follow‐up date. As DAC maintenance was aimed at offsetting relapse or need for salvage therapy and in some patients, salvage was implemented before overt relapse, *time to next treatment (TTNT)* was calculated from the initiation of DAC maintenance to the first salvage regimen or death and censored at last follow‐up. Salvage treatment included the first therapy received after the DAC maintenance in view of molecular relapse, progression, or hematological relapse and included allo‐SCT done directly after DAC maintenance. Molecular relapse‐free survival (mRFS) was calculated for responders in Groups 1 and 2B from attainment of CMR to loss of CMR, hematological relapse or death (whichever was earlier) and for patients in Group 2A from DAC maintenance initiation to similar endpoints mentioned above. mRFS was censored at last follow‐up. Patient characteristics are summarized using frequency (%) for categorical variables and median (range) for continuous variables. Fisher's exact test was used to assess the association between categorical variables. Kaplan–Meier method was used to estimate the probabilities of TTNT and OS. Statistical analyses were performed using GraphPad Prism version 9, GraphPad Software.

## 3 RESULTS

Thirty‐one patients with CBF‐AML [*t*(8;21) = 14 and inv(16) = 17] received DAC as maintenance. The median age of the overall cohort was 57 years (range = 21–78) with a median follow‐up of 59.3 months (range = 13.2–106 months) from DAC initiation. Fifteen patients (48.3%) had persistent qRTPCR positivity (median copies = 0.3, range = 0.01–0.91) after completing a full course (7 cycles) of a FLAG‐based regimen and were subsequently transitioned to DAC (Group 1). Sixteen patients (51.7%) were switched to DAC as they did not complete planned consolidation cycles due to prolonged myelosuppression or other reasons (Group 2). In Group 2, eight patients (50%) had undetectable qRTPCR (Group 2A) and eight patients (50%) had a median positive qRTPCR of 0.1% (range = 0.02%–0.36%) (Group 2B) prior to starting DAC. The median number of DAC cycles were 12 (range = 1–22), 5 (range = 2–18), and 4 (range = 2–13) in Groups 1, 2A, and 2B, respectively.

ACA were present in 16 (52%) of the patients, most common of which were −Y and +8 in four patients each. *KIT* mutations were the most common (10 patients, 32%) followed by *FLT3* (9 patients, 29%, 4 ITD, 4TKD, 1ITD and TKD) and *RAS* mutations (8 patients, 25%). Overall, 21 patients (67.7%) had coexisting mutations (20 with only kinase mutations); 10 from Group 1 (47.6%), 5 from Group 2A (23.9%), and 6 from Group 2B (19.5%).

Patient characteristics and treatment are summarized in Table 1 and response to DAC maintenance and subsequent therapy in Table 2.

**TABLE 1:** Patient characteristics

Characteristics	Group 1 (*N* = 15)	Group 2 (*N* = 16)	
		Group 2A (*N* = 8)	Group 2B (*N* = 8)
	N (%)/median [range]		
Age (years)	50 [29–75]	61 [29–72]	58 [34–78]
Gender			
Male	8 (53.3)	1 (12.5)	3 (37.5)
Follow‐Up (months) after DAC initiation	71.2 [18.6–105.9]	50.0 [21.8–107.5]	22.36 [19.8–68.1]
Cytogenetics			
inv(16)	8 (53.3)	4 (50)	5 (62.5)
t(8;21)	7 (47.7)	4 (50)	3 (37.5)
Hematologic parameters			
Bone marrow blast	1 [0–3]	1 [0–2]	1 [0–4]
WBC (K/μl) × 10^9^/L at start of DAC	4 [1.7–11]	2.5 [1.1–6]	2 [1–6.3]
Hgb (g/dl) at start of DAC	11 [7.4–16.3]	9.9 [7.8–13]	9.2 [7.3–12.2]
Platelets × 10^9^/L at start of DAC	98 [50–244]	53 [27–191]	98 [32–142]
Mutations			
KIT	6 (40)	2 (25)	2 (25)
FLT3	4 (26.6)	4 (50)	1 (12.5)
RAS	5 (33.3)	1 (12.5)	2 (25)
Treatments/PCR			
FLAG cycles	7	4 [3–6]	4 [1–6]
RTPCR at start of DAC	0.03 [0.01–0.91]	0 [0–0.1]	0.1 [0.02–0.36]
DAC cycles	12 [1–22]	5 [2–18]	4 [2–13]

Abbreviations: CBF‐AML, core binding factor acute myeloid leukemia; DAC, decitabine; Hgb, hemoglobin; WBC, white blood count.

**TABLE 2:** Patient outcomes

Characteristics	Group 1 (*N* = 15)	Group 2A (*N* = 8)	Group 2B (*N* = 8)
	*N* (%)/median [range]		
Response			
CMR as best response with DAC	10 (66.7)	8 (100)	2 (25)
Molecular relapse from CMR	2 (20)	2 (25)	2 (100)
Hematological relapse with/ after DAC	1 (6.7)	1 (12.5)	3 (37.5)
Allo‐SCT	4 (26.7)	1 (12.5)	5 (62.5)

Abbreviations: allo‐SCT, allogeneic stem cell transplantation; DAC, decitabine; FLAG, fludarabine; G‐CSF, cytarabine (FLAG)‐based regimen; PCR, polymerase chain reaction.

### 3.1 Response to DAC maintenance

#### 3.1.1 Group 1

Fifteen patients received a median of 12 cycles of DAC maintenance (1–22 cycles). Ten patients (66.7%) cleared the fusion transcript during DAC maintenance and remained in CMR during maintenance. Five patients never attained CMR with DAC and had a positive qRTPCR after DAC maintenance at a median of 0.56% (0.01%–41.42%). Only one patient in this group had a hematological relapse (never attained CMR); this relapse was preceded by molecular progression. Overall, 6 patients needed salvage therapy, at a median of 21.1 months (2.1–36.6 months) after DAC initiation and 10 median cycles of DAC (range = 1–22 cycles). One patient had a late molecular relapse (after 22nd DAC cycle) after having persistent low PCR values of ≤ 0.01% and was thus continued DAC beyond the 12 cycles. He is also the only patient in this group who needed salvage therapy after having attained CMR as his best response.

Amongst the nine patients who did not need salvage therapy, two patients received less than 12 cycles of DAC due to therapy related cytopenia while remaining in persistent CMR and hence DAC was discontinued early.

Two of the six patients who needed salvage therapy proceeded directly to allo‐SCT in view of molecular persistence (never attained CMR with DAC), two received salvage therapy followed by allo‐SCT (one with the delayed molecular relapse and one with hematological relapse) and two patients received salvage chemotherapy without SCT for molecular persistence. At a median follow‐up of 71.5 months (33.6–106 months) after DAC initiation 14 patients (93.3%) are alive with a median estimated OS of 114 months (41.7–115.7 months).

#### 3.1.2 Group 2A

This group had eight patients who had received a median of 4 cycles of FLAG‐based chemotherapy (3–6 cycles) and no PCR positivity at the end of these cycles. The median number of DAC cycles received were 5 (2–18 cycles). Two patients (25%) needed salvage therapy, both for molecular relapse after 5 and 6 cycles of DAC, respectively. Four of the other six patients received less than 12 cycles of DAC due to patient/physician preference (mostly due to recurrent cytopenia with DAC) but without any evidence of relapse at last follow‐up.

At a median follow‐up of 65.1 months (21.8–101.3 months) from DAC initiation, six patients are alive with an estimated OS for the group at 101.9 months (29.1–108.2 months).

#### 3.1.3 Group 2B

There were eight patients belonging to this group, all of whom had received less than the 7 designated cycles (median = 5, range = 1–6 cycles) of FLAG‐based chemotherapy, and had persistent median qRTPCR positivity at 0.1% (range = 0.02%–0.36%). The median cycles of DAC received in this group were 4 (2–13 cycles). Two patients (25%) attained a CMR, and one patient had a 2‐log reduction with DAC maintenance, but all three subsequently had a molecular relapse. All the patients in this group were initiated on a second line of therapy which was also the reason for early termination of DAC maintenance (only one patient continued DAC beyond 12 cycles). Five of them were able to proceed to an allo‐SCT (four after salvage chemotherapy and one directly). The median PCR prior to initiation of the second line of therapy was 35.34% (0.02%–77.48%). At a median follow‐up of 26.8 months (13.2–58.8 months) from DAC initiation, three patients were alive with a median estimated OS of 34 months (20.8–68.8 months).

### 3.2 Flow cytometry MRD

Flow cytometry based MRD sensitive for the detection of 1 leukemia cell in 10^4^ cells were analyzed for all patients prior to DAC therapy initiation and at all‐time points with BM qRTPCR evaluation. Amongst the 23 patients in Groups 1 and 2B who had a PCR positivity at > 0.01%, only 3 patients had an MRD positive by flow cytometry. At the post‐DAC assessment timepoint, amongst 13 patients with a positive PCR, only 7 patients had a positive flow cytometry. Flow cytometry MRD assessment was however able to identify relapse in one patient with non‐CBF relapse who remained PCR negative. The Pearson corelation coefficient (*R*) between concomitant pre‐DAC PCR and flow MRD values was .0004 (95% confidence interval [CI] = −0.037 to 0.032) and 0.002 (95% CI = −0.34 to 0.42) for post‐DAC values. Negative qRTPCR was reflected in negative flow MRD, but at any higher value the correlation was poor. Thus, PCR‐based MRD assessment was more sensitive than flow‐based assessment in our patients.

### 3.3 Survival and TTNT

We assessed TTNT as an important endpoint in our study, as DAC maintenance, by virtue of clearing MRD, should preclude or delay need for salvage therapy.

**FIGURE 1:**
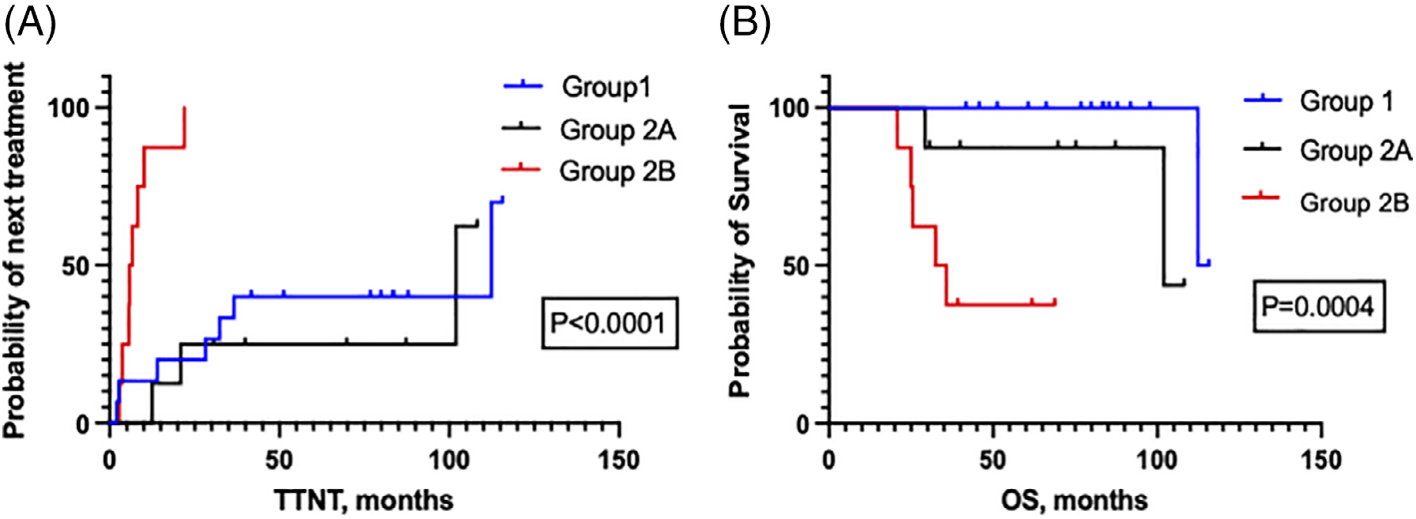
(A and B) Kaplan–Meier estimates of TTNT and OS amongst the three groups. Patients in Group 2B had a significantly short time to next treatment with DAC maintenance and overall survival compared to patients in Groups 1 and 2A. DAC, decitabine; OS, overall survival; TTNT, time to next treatment [Color figure can be viewed at wileyonlinelibrary.com]

The median TTNT was 112.3 months for Group 1 versus 101.9 months for Group 2A and 6.3 months for Group 2B (*p* log rank < .0001 between groups, *p* log rank = .08 for Groups 1 vs. 2) (Figures 1A and S1A). For the 20 patients with a best response of CMR (12 from Groups 1 and 2B who attained CMR with DAC maintenance and 8 from Group 2A who started with CMR on DAC), the estimated mRFS was 94 months (96.5 months in Group 1 + 2B and 93.9 months in Group 2A, *p* = .7) (Figure S2A,B). At a median follow‐up of 59.3 months from DAC initiation for the entire cohort, the Kaplan–Meier estimates for OS was 114 months for Group 1 versus 101.9 months for Group 2A and 34 months for Group 2B (*p* log rank = .0004 between groups, *p* log rank = .004 for Group 1 vs. Group 2) (Figures 1B and S1B).

**FIGURE 2:**
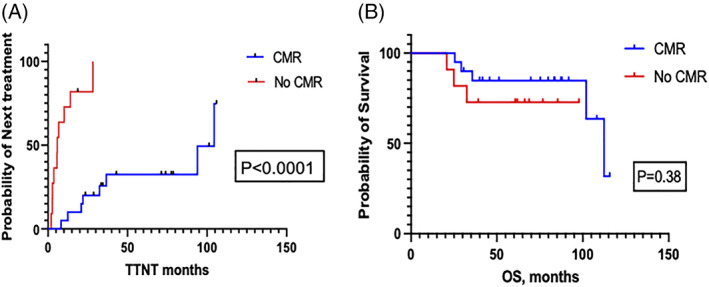
(A and B) TTNT and OS estimates of patients who attained CMR as their best response with DAC versus those who did not. Patients who attained CMR with DAC had a longer time to next treatment compared to those who did not attain CMR, however, there were no difference in OS between these groups. DAC, decitabine; OS, overall survival; TTNT, time to next treatment [Color figure can be viewed at wileyonlinelibrary.com]

The median TTNT in patients who attained a best response of CMR with DAC was 112.4 months compared to 5.8 months in those who never attained a CMR (hazard ratio [HR] = 0.16, 95% CI = 0.04–0.54). The median estimated OS in patients with CMR was 116.3 months versus not reached (NR) for patients who never attained a CMR (*p* = .37) (Figure 2A,B).

**FIGURE 3:**
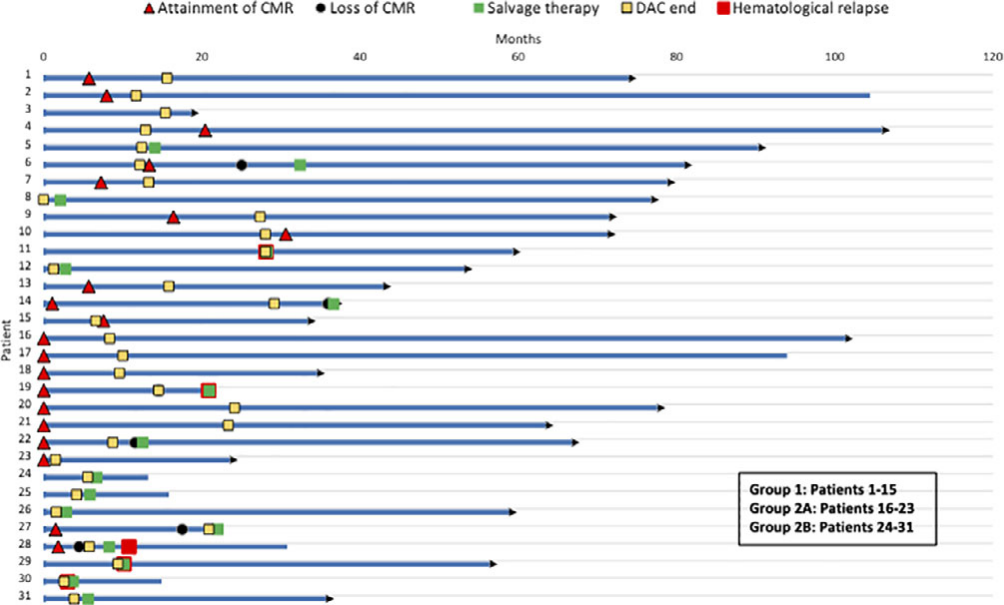
Swimmer's plot of patients in the three groups depicting time to attainment of CMR from DAC initiation, molecular relapse (loss of CMR), hematological relapse and initiation of salvage therapy. Patients with arrowheads at end are alive at last follow‐up. Time point “0” on the *X*‐axis is the time of DAC maintenance initiation. DAC, decitabine [Color figure can be viewed at wileyonlinelibrary.com]

Of the 23 patients in Group 1 and 2 B (all had a positive pre‐DAC PCR), 12 patients (52.2%) attained CMR as their best response to DAC maintenance, at a median of 7.4 months from DAC initiation (1.5–30 months). Four of these patients (33%) had a molecular relapse (PCR ≥ 0.01%) at 2.6, 11.7, 16, and 31.4 months from attainment of CMR, but there were no hematological relapses. Amongst the 11 patients (47.8%) from Group 1 + 2B who never attained a CMR while on maintenance, 3 had a hematological relapse. All patients with relapses or molecular persistence were subsequently transitioned to a salvage therapy (Figure 3: Swimmer's plot). The median estimated TTNT for patients who attained CMR in these two groups were 57.1 versus 5.8 months in those who did not attain CMR (*p* log rank < .001).

We further analyzed the possibility of attaining a CMR with DAC maintenance in all the patients based on their pre‐DAC PCR values (≤ 0.01% = low and > 0.01% = high). All 13 patients with low PCR positivity (8 patients with PCR = 0% and 5 patients with PCR = 0.01%) attained CMR (PCR < 0.01%), compared to 7 of 18 patients in the high PCR group (*p* = .0004). In patients with low pre‐DAC PCR the median TTNT was 112.4 months compared to 12 months in patients with high pre‐DAC PCR (HR = 0.31, 95% CI = 0.12–0.8) and median OS was 112.4 months versus NR (HR = 0.6 [NS], 95% CI = 0.16–2.5) (Figure S3A,B).

### 3.4 Impact of cytogenetics, KIT, and kinase mutations

Based on the CBF types, 11 patients (64.7%) with inv(16) and 9 patients (64.2%) with t(8;21) attained CMR as their best response. Eight patients in Group 1 and two patients each in Groups 2A and 2B had baseline ACA. There was no difference in the rates of CMR attainment in these groups based on the presence of ACA (*p* = .9). *KIT* mutations were present in 10 patients at baseline (six in Group 1, two in Groups 2A and 2B, respectively). There was no difference in the TTNT or OS between patients who had a *KIT* versus no *KIT* mutation; 18 versus 102 months (*p* = .2) and 112.4 months versus NR (*p* = .4), respectively. Overall, 20 patients (64.5%) had a kinase mutation (*FLT3 ± RAS ± KIT*); 11 (55%) of them had CMR with DAC as their best response compared to 9 patients (81%) without kinase mutations who attained CMR (*p* = .25). There was no difference in the median estimated TTNT or OS between patients who had kinase mutation versus those who did not; 21.9 versus 36.6 months (HR = 1.3, 95% CI = 0.52–3.4) and 102.2 versus 112.3 (HR = 1.1; 95% CI = 0.27–4.5).

### 3.5 Stem cell transplantation

In our cohort, 10 patients (32.2%) had to subsequently undergo an allo‐SCT (4 patients in Group1, 1 patient in Group 2A, and 5 patients in Group 2B). This included three patients who were directly taken up for allo‐SCT in view of molecular persistence (0.02%, 0.12%, and 0.17%) despite DAC maintenance. All three of them attained post‐SCT CMR and continues to remain alive at median of 50.5 months after SCT. The remainder seven patients received interim salvage therapy before SCT; two of them died, 42 and 274 days after allo‐SCT, respectively. At a median follow‐up of 42.9 months (1.3–74.7 months) after SCT, eight patients are alive in PCR negative CR, with a median OS not reached in the survivors.

## 4 DISCUSSION

In the present study we have shown that DAC can be a possible maintenance option in patients with CBF‐AML who are unable to complete the desired duration of consolidation therapy and/or have persistent PCR transcripts post consolidation therapy. The aim of DAC maintenance was to delay or prevent relapse. Patients who had a best response of CMR with or before DAC had a good median mRFS of 94 months. We used TTNT as an important outcome in our study as this captured the important duration of time the maintenance regimen was able to delay the need for salvage therapy or prevent death and thus be a surrogate for RFS. The hematological RFS was difficult to capture in all patients, as in a significant number of patients, interventions were done before overt relapse. Attainment of CMR with DAC maintenance in patients who have persistent PCR positivity post consolidation therapy or maintenance of CMR in patients in whom intensive consolidation therapy was truncated, significantly delayed TTNT.

The importance of MRD monitoring in CBF‐AML has been well defined and discussed before.^15^, ^29^, ^30^, ^31^, ^32^ The ability to monitor molecular MRD with transcript specific qRTPCR allows accurate assessment of remission status and prediction of impending morphological relapse in CBF‐AML as has been shown in the UK MRC AML 15 trial and by other groups.^12^, ^33^ Allo‐SCT is one possible intervention for persistent positive qRTPCR or molecular relapse (MRD reoccurrence without hematological relapse) with or without any intervening salvage therapy and is recommended by the European Leukemia Network 2017 guidelines for treatment of AML.^34^, ^35^ However, donor availability, patient preference and patient‐related factors including age, performance status, and organ dysfunction may be potential barriers to timely and safe allo‐SCT.^36^ Given the risks of treatment related morbidity and mortality with allo‐SCT, other therapeutic options need to be explored which can be guided by the depth of MRD persistence, are more tolerable, and easy to administer. Recent approvals of oral HMAs make this option attractive.

HMA‐based maintenance approach has received recent approval in AML with high risk of relapse, but these studies did not include patients with CBF‐AML. Earlier, we reported on the activity of HMA as maintenance therapy in a smaller cohort of patients with CBF‐AML and with short follow‐up.^25^ Eleven of twelve patients who maintained remission with HMA had a reduction in qRTPCR either after the first or second cycle of HMA. In that cohort, patients were treated with either DAC or AZA for persistent low qRTPCR positivity after various induction/consolidation regimens, including allo‐SCT and salvage therapy. Here, we report on a larger and a more homogenous group treated upfront with only FLAG‐based regimens and DAC alone as the maintenance.

The importance of completing all scheduled cycles of induction/consolidation chemotherapy for long‐term survival in CBF‐AML cannot be overemphasized.^4^ In the MRC cohort, CBF‐AML patients who completed all FLAG‐ and HDAC‐based induction/consolidations had stellar outcomes^37^ In our cohort also on comparing TTNT between Groups 1 and 2; there was a significant difference in the OS and there was a trend toward significance in TTNT between these two groups. On a three‐way comparison amongst the groups (with Groups 1 and 2A superimposing), the TTNT and OS were significantly shorter for Group 2B.

To stratify the importance of depth of remission after chemotherapy and how DAC maintenance would affect that, we assessed the TTNT and OS of patients with low and high PCR values and found that low pre‐DAC PCR (≤0.01%) improved the possibility of CMR (< 0.01%) and subsequently increased the TTNT across the three groups. Thus, maximum benefit of DAC maintenance can be achieved in patients who have a low baseline postconsolidation PCR.

Overall, the best benefit of the DAC maintenance was in patients who had received all the cycles of therapy and had a low burden of pre‐DAC PCR. Thus, patients with higher PCR burden after complete or attenuated cycles of chemotherapy should be considered for allo‐SCT consolidation over DAC maintenance, and further studies will be needed to define the PCR cutoff for that decision. In the present study, all patients from Group 2B needed salvage therapy after DAC maintenance; a group with the highest risk given their inability to complete desired consolidation cycles of chemotherapy and having pre‐DAC PCR positivity at >0.01%.

This is the largest study with a significant follow‐up of patients with CBF‐AML who were initiated on DAC maintenance in a structured manner. However, due to relatively small number in individual subsets, it precluded the stratified analysis of the effect of mutations (other than KIT) on the response to DAC and outcomes. Though in our study the presence of KIT mutation did not negatively affect the attainment of CMR with DAC and TTNT, CBF‐AML patients with KIT mutations are conventionally considered to be high risk and often transplanted in first remission.^38^, ^39^ The variable number of DAC cycles administered and the heterogeneous time points at which salvage therapy was initiated are limitations of our study. Thus, it is difficult from this study to recommend the ideal duration of DAC maintenance, though at our institution we administer usually for 12 cycles. Importantly majority of the patients were identified and received salvaged after molecular relapse and prior to overt hematological relapse.

Benefit of allo‐SCT for patient with suboptimal qRTPCR response has been reported but may not be the most suitable option for all. The risk of delaying allo‐SCT lies in the possibility of rapid overt relapses while on DAC maintenance. For patients considered to be at high risk for such an event based on qPCR cut‐offs, are better off being evaluated early for SCT. Randomized trials designed to compare the benefit of predefined duration of DAC maintenance versus allo‐SCT for specific postconsolidation PCR cut‐offs will help to make more informed decisions and help better identification of CBF‐AML patients who should be transplanted earlier as they are poised to benefit less from DAC maintenance. The importance of completing all courses of chemotherapy in conjunction with PCR MRD transcript levels also need to be studied to understand the importance of each in long‐term survival.

## 5 CONCLUSION

CBF‐AML patients who are unable to complete all planned consolidation therapy or have persistent disease specific transcripts detectable at low levels via qRTPCR benefit from DAC maintenance in terms of long mRFS and salvage treatment free remission or death. Larger studies will be required to designate the subset of patients who have the maximal chance of benefit from this approach.

## CONFLICT OF INTEREST

Tapan Kadia: Research grants—Amgen, Ascentage, Astellas, AstraZeneca, BMS, Cellenkos, Pulmotech, Genfleet. Personal fees from—Agios, Cure, Daichi Sankyo, Genzyme, Liberum, Novartis, Sanofi‐Aventis. Research grants and personal fees—Abbvie, Genetech, Jazz Pharmaceuticals, Pfizer. Naveen Pemmaraju: Research grants—Novartis, Stemline Therapeutics, Samus Therapeutics, Abbvie, Cellectics, Affymetrix/Thermo Fisher Scientific, Daiichi Sankyo, Plexxikon, MustangBio. Honoraria—Incyte, Novartis, LFB Biotechnologies, Stemline Therapeutics, Celgene, Abbvie, MustangBio, Roche Molecular Diagnostics, Blueprint Medicines, DAVA Pharmaceuticals, Springer Science+Business Media LLC, Aptitude Health, NeoPharm, CareDX. Consulting or advisory role—Blueprint Medicines, Pacylex Pharmaceuticals Inc., Immunogen, Mristol Myers Squibb, Clearview Healthcare Partners, Astellas Pharma US Inc., Protagonist Therapeutics, Triptych Health Partners, CTI BioPharma Corp. Travel/accommodation expenses—Stemline Therapeutics, Celgene, Abbvie, DAVA oncology, MustangBio. Courtney DiNardo: Research grants—Abbvie, Agios/Servier, Astex, Calithera, Celgene/BMS, Cleave, Daiichi‐Sankyo, ImmuneOnc, Loxo. Honoraria/consulting fees—Abbvie, Agios/Servier, Astellas, Celgene/BMS, Cleave, Foghorn, Genentech, Novartis, Notable Labs, Takeda. Other authors declare no conflict of interest.

## Supporting information


**Figure 1A and 1B** Compared to patients in Group 1, patients in group 2 had a shorter OS but no difference in the time to next treatment with DAC maintenance.
**Figure 2A and B:** Kaplan Meier estimates of molecular relapse free survival: a) 94 months in the whole cohort b) 96.5 month in group 1 + 2B vs. 93.9 months in group 2A (p‐0.7)
**Figure 3A and B:** Patients with low pre‐DAC PCR (≤0.1%) had a longer TTNT (112.4 vs. 12 months) with DAC maintenance but not OS (112.4 months vs. not reached) compared to those who had high pre‐DAC PCR (>0.1%)

## Data Availability

Data available on request from the authors.
