# Supplementary material for: Activity of decitabine as maintenance therapy in core binding factor acute myeloid leukemia
Source: Am J Hematol. 2022 Feb 22;97(5):574–82. doi: 10.1002/ajh.26496 (PMC9303262; doi:10.1002/ajh.26496)
Supplement: Supplementary file 1 — Figure 1A and 1B Compared to patients in Group 1, patients in group 2 had a shorter OS but no difference in the time to next treatment with DAC maintenance. Figure 2A and B: Kaplan Meier estimates of molecular relapse free survival: a) 94 months in the whole cohort b) 96.5 month in group 1 + 2B vs. 93.9 months in group 2A (p‐0.7) Figure 3A and B: Patients with low pre‐DAC PCR (≤0.1%) had a longer TTNT (112.4 vs. 12 months) with DAC maintenance but not OS (112.4 months vs. not reached) compared to those who had high pre‐DAC PCR (>0.1%) [file AJH-97-574-s001.docx]

**Supplementary figure 1**

| **Supplementary Figure 1A**  **** | **Supplementary Figure 1B**  **** |
| --- | --- |

**Supplementary Figure 1A and 1B:** Compared to patients in Group 1, patients in group 2 had a shorter OS but no difference in the time to next treatment with DAC maintenance.

**Supplementary figure 2**

| **Supplementary figure 2A**   | **Supplementary figure 2B**   |
| --- | --- |

**Supplementary figure 2:** Kaplan Meier estimates of molecular relapse free survival: a) 94 months in the whole cohort b) 96.5 month in group 1+2B vs. 93.9 months in group 2A (*p*-0.7)

**Supplementary figure 3**

| **Supplementary figure 3A**  **** | **Supplementary figure 3B**  **** |
| --- | --- |

**Supplementary Fig. 3A and 3B:** Patients with low pre-DAC PCR (≤0.1%) had a longer TTNT (112.4 vs. 12 months) with DAC maintenance but not OS (112.4 months vs. not reached) compared to those who had high pre-DAC PCR (>0.1%)
